# Povidone-Iodine Versus Homemade Salt/Soda Mouthwash for the Prophylaxis of Radiation-Induced Oral Mucositis in Head and Neck Cancer: A Quasi-experimental Study

**DOI:** 10.7759/cureus.116545

**Published:** 2026-09-19

**Authors:** Dipok Saha, Nazirum Mubin, Tannima Adhikary, Rukhshana Rabbani, Mukti Datta, Nahid Naznin Rinky, Mohammad Saiful Islam Pathan, ATM Shazzaeatur Rahaman, Aliya Shahnaz, Qazi Mustaq Hussain

**Affiliations:** 1 Department of Radiotherapy, Sirajganj Medical College Hospital, Sirajganj, BGD; 2 Department of Radiotherapy, Dhaka Medical College Hospital, Dhaka, BGD; 3 Clinical Oncology, Imperial College London, London, GBR; 4 Department of Radiotherapy, Mymensingh Medical College Hospital, Mymensingh, BGD; 5 Department of Clinical Oncology, Labaid Cancer Hospital and Super Speciality Centre, Dhaka, BGD

**Keywords:** head and neck cancer, oral mucositis, povidone-iodine, quasi-experimental study, radiotherapy, salt/soda mouthwash, sodium bicarbonate

## Abstract

Background

Radiation-induced oral mucositis (RIOM) is a common acute toxicity of radiotherapy for head and neck cancer and may compromise treatment delivery. Povidone-iodine is commonly used for prophylaxis at our centre, whereas a homemade salt/soda mouthwash is an inexpensive alternative. This study compared these two rinses for preventing RIOM.

Methods

This prospective quasi-experimental study enrolled 110 adults with histologically confirmed head and neck cancer receiving conventional two-dimensional external-beam radiotherapy (60-66 Gy over 6-6.5 weeks) at Dhaka Medical College Hospital, Bangladesh. Patients were assigned by odd/even registration number to the 1% povidone-iodine or salt/soda mouthwash group (n=55 each), which they used every six hours from the first day of radiotherapy until 14 days after completion. Oral mucositis was graded weekly and at two and four weeks after radiotherapy using the World Health Organization (WHO) scale, while oral pain was assessed using a 10-cm visual analogue scale. The primary outcome was WHO grade ≥3 mucositis; secondary outcomes included its duration, oral pain, analgesic and antifungal use, and treatment interruption or discontinuation.

Results

WHO grade ≥3 mucositis first appeared at week 3 and was less frequent with salt/soda than povidone-iodine at week 3 (0/55 (0%) vs. 10/55 (18.2%), p=0.001), week 4 (4/55 (7.3%) vs. 14/55 (25.5%), p=0.010), and week 5 (10/55 (18.2%) vs. 20/55 (36.4%), p=0.032). After Bonferroni correction, the week 3 difference remained significant, while the week 4 difference was at the adjusted threshold. Overall severe mucositis was less frequent with salt/soda (43.6% vs. 60.0%; absolute risk difference −16.4%, 95% CI −34.8 to 2.1), although the difference was not statistically significant (p=0.086). Median duration of severe mucositis was similar (16.5 vs. 20.0 days, p=0.21). Oral pain was lower with salt/soda at weeks 3 and 4 (p=0.001 and p=0.006). Antifungal use was higher with salt/soda (83.6% vs. 63.6%, p=0.017), while treatment interruption and discontinuation occurred only with povidone-iodine.

Conclusion

In this study, salt/soda mouthwash showed an early trend toward lower severe RIOM and oral pain than povidone-iodine, but overall incidence and duration did not differ significantly. Because allocation was predictable, baseline imbalances were present, and analyses were unadjusted, these findings should be considered exploratory. Salt/soda may be a practical low-cost option in resource-limited settings, but adequately powered randomized multicentre trials are required for confirmation.

## Introduction

Head and neck cancers constitute a substantial global health burden, with marked geographical variation in incidence and mortality [1]. Radiotherapy remains a core definitive or postoperative treatment for many patients and is frequently combined with systemic therapy in locally advanced disease [2]. Radiation-induced oral mucositis (RIOM) is one of the most frequently observed acute toxicities when the oral or oropharyngeal mucosa is included in the treatment volume. A recent meta-analysis estimated a pooled RIOM prevalence of 94%, while a contemporary intensity-modulated radiotherapy cohort study documented a clinically important burden of severe mucositis, analgesic use, feeding-tube placement, and treatment interruption [3,4].

RIOM is a dynamic biological process rather than a simple consequence of epithelial cell loss. Current models describe overlapping phases of initial DNA and tissue injury, reactive oxygen species generation, activation and amplification of pro-inflammatory signalling, ulceration with secondary biological interactions, and eventual healing [5,6]. During the ulcerative phase, disruption of the mucosal barrier can increase local pain and susceptibility to infection. Alterations in the oral microbiome have also been associated with delayed mucosal healing after radiotherapy, although the direction and clinical significance of this relationship remain uncertain [7]. These mechanisms provide a rationale for interventions that reduce inflammation, preserve tissue integrity, support oral hygiene, or modify the local oral environment.

The risk and severity of RIOM are heterogeneous. Treatment-related determinants include the irradiated mucosal volume and dose distribution, total dose, fractionation schedule, and concurrent systemic therapy [4,5,8]. Patient- and disease-related characteristics, including tumour subsite, baseline nutritional status, oral hygiene, salivary function, smoking, comorbidity, and laboratory parameters, may also influence susceptibility; however, the direction and strength of several associations are inconsistent across studies [9,10]. This heterogeneity complicates comparison among mucositis trials and makes balanced allocation and careful reporting of potential confounders particularly important.

The consequences extend beyond visible ulceration. Severe RIOM causes oral pain, dysphagia, odynophagia, and difficulty speaking, eating, drinking, and maintaining oral hygiene [4,7]. Reduced oral intake can contribute to dehydration, weight loss, and deterioration of nutritional status, while severe symptoms may require opioid analgesia, enteral feeding, or hospital admission [11-14]. Prospective and systematic evidence indicates that severe mucositis is associated with poorer health-related quality of life during treatment [11,12]. Nutritional review data further support early assessment and intervention as part of multidisciplinary supportive care, even though nutritional support is not a substitute for mucositis-directed prevention [13].

Preventing RIOM remains challenging because no single intervention is appropriate for every oncology setting. The Multinational Association of Supportive Care in Cancer and International Society of Oral Oncology (MASCC/ISOO) guidelines place structured basic oral care at the foundation of prevention and management [15]. Systematic reviews have evaluated anti-inflammatory agents, antimicrobials, mucosal coating agents, analgesics, photobiomodulation, and natural products, but study populations, treatment regimens, outcome scales, and intervention protocols vary substantially [16-18]. A recent expert consensus similarly emphasised comprehensive oral assessment and care across radiochemotherapy [19]. Consequently, recommendations for individual agents are linked to specific cancer-treatment contexts and the certainty of the available evidence rather than to a universal hierarchy of mouthwashes.

Among better-studied approaches, photobiomodulation reduces the incidence, severity, and pain of oral mucositis, although access to equipment, trained personnel, and standardised dosimetry may limit routine implementation, according to a recent meta-analysis of randomised trials [20]. Benzydamine is recommended in selected radiotherapy settings, and a recent phase IV study reported high treatment compliance, but its single-arm design precluded definitive comparative inference [15,21]. In contrast, evidence for bland sodium bicarbonate-containing rinses is heterogeneous and does not establish superiority over other agents [22]. A multicentre practice survey also demonstrated considerable variation in routine RIOM prevention and treatment, illustrating the persistent gap between guidelines and real-world supportive care [23].

At our centre, povidone-iodine mouthwash is commonly prescribed for RIOM prophylaxis because povidone-iodine has broad antimicrobial activity [24]. Contemporary mucositis guidelines, however, do not identify it as a preferred prophylactic rinse [15,17]. Recent oral-exposure studies show that iodine can be absorbed or ingested during intra-oral povidone-iodine use: one short-exposure study found increased serum and urinary iodine without altered free thyroxine, while another documented iodine ingestion that could exceed the tolerable upper intake level [25,26]. Oral candidiasis may occur during head and neck radiotherapy and can coexist with mucositis, but clinical diagnosis and antifungal use do not necessarily establish microbiological infection [27]. A homemade salt/soda rinse, prepared from sodium chloride and sodium bicarbonate, is bland, requires no commercial product, and avoids iodine exposure; these features may be relevant in resource-limited settings.

Contemporary reviews and practice data do not identify a recent direct head-to-head comparison of povidone-iodine and homemade salt/soda mouthwash for RIOM prophylaxis, and the broader evidence on mouth rinses remains heterogeneous [16,17,22,23]. A direct comparison was therefore warranted to inform local practice and generate evidence for a low-cost intervention. We conducted a prospective quasi-experimental study comparing 1% povidone-iodine with homemade salt/soda mouthwash in patients receiving conventional radiotherapy for head and neck cancer. The objectives were structured hierarchically. The primary objective was to compare the incidence of WHO grade ≥3 oral mucositis between the two rinses during radiotherapy and the first four weeks afterwards [28]. The secondary objectives were, in order, to compare the duration of WHO grade ≥3 mucositis among affected patients, patient-reported oral pain, the use of analgesics and antifungals, and the impact on radiotherapy delivery. Oral pain was placed among the secondary, patient-reported outcomes rather than among the primary outcomes because pain intensity is a subjective experience that differs between individuals. Because individual allocation followed a predictable odd/even sequence and no adjusted analysis was planned, the study was designed to describe associations and trends between rinse type and mucositis outcomes rather than to establish a causal or comparative-effectiveness claim.

## Materials and methods

Study design and setting

This single-centre, prospective, quasi-experimental study was conducted in the Department of Radiotherapy, Dhaka Medical College Hospital, Dhaka, Bangladesh, over a period of one year from November 2019 to October 2020.

Participants

Eligible patients were adults aged 20-70 years with histologically confirmed head and neck cancer scheduled for external-beam radiotherapy using a two-dimensional conventional technique on a cobalt-60 machine, with a treatment volume including the oral and/or oropharyngeal mucosa, receiving 200 cGy daily to a total of 60-66 Gy over 6-6.5 weeks, and an Eastern Cooperative Oncology Group (ECOG) performance status of 0-2. Patients were excluded if they had pre-existing oral mucositis from other causes (prior chemotherapy or radiotherapy, bacterial or fungal infection), pre-existing mucosal atrophy or xerostomia, iodine hypersensitivity, uncontrolled diabetes mellitus, were pregnant or lactating, were receiving concurrent chemoradiation, were enrolled in another clinical trial, or were unable to attend follow-up. Eligible patients were recruited by convenience sampling from those attending the department during the study period.

Sample size

The sample size was based on the incidence of WHO grade ≥3 mucositis reported in an earlier randomised comparison of prophylactic mouthwashes, including povidone-iodine and salt/soda, in patients receiving radiotherapy for head and neck cancer [29]. Assuming severe mucositis in 50% and 24% of the salt/soda and povidone-iodine arms, respectively (standardized difference 0.53), 110 patients were required to provide 80% power at a two-sided significance level of 0.05. These event rates were taken directly from that published trial and were not derived from local audit data, and the assumption anticipated a higher rate of severe mucositis in the salt/soda arm than in the povidone-iodine arm. The direction of the difference actually observed was the opposite of the direction assumed at the planning stage (60.0% with povidone-iodine and 43.6% with salt/soda over the whole treatment period). The calculation should therefore be read as a pre-study planning assumption rather than as a validated local estimate. In practice, the sample provided adequate power only for a difference of about 26 percentage points and was underpowered for the smaller whole-period difference of 16.4 percentage points that was observed, and it was not sized to support adjustment for confounding or formal trend modelling.

Group allocation and interventions

Patients were enrolled sequentially and assigned according to the parity of their registration numbers: odd numbers to arm A and even numbers to arm B. Before enrolment began, a lottery assigned the interventions to the arm labels, with povidone-iodine allocated to arm A and salt/soda to arm B; the lottery did not randomize individual patients. Because individual assignment followed a predictable odd/even sequence, the study was classified as quasi-experimental. Arm A rinsed with 15 mL of 1% povidone-iodine mouthwash and arm B with 15 mL of salt/soda mouthwash, every six hours, from the first day of radiotherapy until 14 days after its completion. Patients were instructed to rinse the oral cavity and oropharynx for 30 seconds at each application. A 1% povidone-iodine mouthwash of a single commercial brand was used for every patient in arm A. The salt/soda mouthwash was prepared by dissolving one teaspoon (~5 g) of table salt and one teaspoon (~5 g) of baking soda in 500 mL of mineral water and was supplied fresh daily. Allocation was neither concealed nor blinded. Neither patients nor assessors could be blinded because the two rinses differ in taste, colour, and smell, and no matched placebo or taste-masked preparation was available at the study site.

Oral and dental assessment and background care

Before radiotherapy began, every patient in both arms underwent a thorough oral and dental examination to exclude pre-existing abnormalities, and patients who required dental extraction did not start radiotherapy until two weeks after successful extraction. Apart from the allocated mouthwash, no other prophylactic rinse, mucosa-directed agent or photobiomodulation was given in either arm. Beyond this pre-treatment assessment, background oral-care practices such as toothbrushing, dietary modifications, and continued use of tobacco or betel quid during treatment were not standardised by a written protocol and were not recorded, so they could not be compared between the arms.

Radiotherapy technique and planning

Radiotherapy was delivered with a single technique in both arms. Every patient was treated with two-dimensional conventional external-beam radiotherapy on a cobalt-60 machine, and no patient in either arm received three-dimensional conformal radiotherapy, intensity-modulated radiotherapy (IMRT) or volumetric modulated arc therapy (VMAT), so the treatment modality itself could not be imbalanced between the arms. Treatment planning was manual and was based on wire and marker simulation with plain radiography; no computed tomography-based planning was used. Fields were arranged either as a unilateral single field or bilateral parallel-opposed fields, and every patient received 200 cGy per fraction to a total dose of 60 or 66 Gy over 6-6.5 weeks. Because planning was two-dimensional and manual, no mucosal dose-volume histogram could be generated, and the volume of oral and oropharyngeal mucosa receiving a low or intermediate dose could not be quantified in either arm; the mucosa lying within the treated fields therefore received a dose close to the prescribed dose in both groups. This is an important difference from IMRT and VMAT, which produce a more conformal dose distribution with greater mucosal sparing, and it is the reason why the present results should not be extrapolated to modern conformal practice.

Outcomes and assessment

The primary outcome was the incidence of WHO grade ≥3 oral mucositis. The secondary outcomes were the duration of WHO grade ≥3 mucositis among affected patients, patient-reported oral pain, the need for analgesics (opioid or non-opioid) and antifungals, and the impact on treatment (continued, interrupted, or discontinued). Oral pain was pre-specified as a patient-reported secondary outcome rather than as a primary outcome because pain intensity is a subjective experience that varies between individuals and is influenced by tumour site, analgesic exposure, candidiasis, and individual reporting style.

Oral mucositis was graded weekly during radiotherapy and at two and four weeks after completion using the WHO oral mucositis scale (grade 0, no mucositis; grade 1, erythema and soreness; grade 2, ulcers with ability to eat solids; grade 3, ulcers requiring a liquid diet because of mucositis; grade 4, ulcers with alimentation not possible because of mucositis) [28]. A full oropharyngeal examination was carried out at every visit using a head light, a tongue depressor, and surgical gloves, and the highest grade observed at any site was recorded as the grade for that visit. Grading was performed by the investigator at the follow-up clinic. Assessors were not blinded to the allocated rinse, no independent duplicate grading was undertaken, and inter-observer agreement was therefore not assessed. Oral pain was recorded at the same visits by the patient, who made a handwritten mark on a 10-cm visual analogue scale (VAS) representing a continuum between “no pain” and “worst pain”.

Need for analgesic use and need for antifungal use were recorded as management outcomes at each visit. Analgesia was mainly oral ibuprofen, a non-steroidal anti-inflammatory drug, and the antifungal used was oral nystatin suspension. Both were prescribed by the treating physician on clinical grounds, and no protocol-defined threshold of pain score or mucositis grade was applied to either decision. Fungal culture and microscopy were not performed at any visit, so antifungal prescription represents a clinical management decision and not microbiologically confirmed candidiasis. Radiotherapy was likewise interrupted or discontinued at the discretion of the treating physician, with no protocol-defined stopping rule based on mucositis grade.

Statistical analysis

Continuous variables are summarized as means ± standard deviations (SDs) or median with interquartile range (IQR), and categorical variables as frequencies and percentages. Groups were compared using the Mann-Whitney U test (age), the Pearson chi-square or Fisher’s exact test (categorical variables and mucositis grades), the independent-samples median test (duration and pain scores), the z-test for proportions (analgesic and antifungal use), and Fisher's exact test (treatment interruption and discontinuation). All between-group comparisons were unadjusted. A two-sided p<0.05 was considered statistically significant. Analyses were performed in IBM SPSS Statistics version 22 (IBM Corp., Armonk, NY, USA); figures were reproduced directly from the primary dataset. Effect sizes for the binary outcomes are additionally reported as absolute risk differences with 95% confidence intervals, and as risk ratios with 95% confidence intervals where both arms contained at least one event; a risk ratio could not be estimated where one arm had no events. No multivariable, stratified, or propensity-based adjustment was performed. With 110 patients and 57 events of WHO grade ≥3 mucositis in total, and with several imbalanced baseline characteristics, the data could not support stable adjustment for indication-dependent confounding or formal modelling of a trend across the treatment weeks, and any such analysis would have been unstable and potentially misleading; the comparisons are therefore presented as unadjusted and the week-specific results as exploratory. Because WHO grade ≥3 mucositis was compared at five separate time points, the week-specific comparisons were also interpreted against a Bonferroni-corrected threshold of p<0.010; both the nominal and the corrected interpretations are reported.

Ethics

The study was conducted in accordance with the principles of the Declaration of Helsinki. The study protocol was approved by the Ethical Review Committee of Dhaka Medical College (approval no. ERC-DMC/ECC/2019/323), and written informed consent was obtained from every participant before enrolment.

## Results

Among the 110 patients, 55 were assigned to each arm. The mean age was 56.22 ± 10.71 years in the povidone-iodine arm and 53.45 ± 11.45 years in the salt/soda arm, with no significant difference in age (p=0.166) or sex distribution (p=0.323). Socio-economic status differed significantly between the arms (p<0.001): poor patients were more common in the salt/soda arm (41 (74.5%) vs. 14 (25.5%)), whereas middle-class patients were more common in the povidone-iodine arm (39 (70.9%) vs. 14 (25.5%)). Educational level also differed significantly (p=0.010), with a higher proportion of illiterate patients in the salt/soda arm (20 (36.4%) vs. 6 (10.9%)) (Table 1).

**Table 1:** Baseline demographic characteristics of the study participants by treatment arm (n=110) Data are presented as n (%) unless otherwise indicated; age is presented as mean ± standard deviation (SD) and as median (range). A two-sided p<0.05 was considered statistically significant. ^a^Mann-Whitney U test ^b^Pearson chi-square test SD, standard deviation; SSC, secondary school certificate; HSC, higher secondary certificate

Variable	Povidone-iodine (n=55)	Salt/soda (n=55)	p-value
Age (years)			0.166^a^
Mean ± SD	56.22 ± 10.71	53.45 ± 11.45	
Median (range)	55 (21–70)	55 (31–70)	
Sex, n (%)			0.323^b^
Male	47 (85.5)	43 (78.2)	
Female	8 (14.5)	12 (21.8)	
Socio-economic status, n (%)			<0.001^b^
Poor	14 (25.5)	41 (74.5)	
Middle class	39 (70.9)	14 (25.5)	
Upper class	2 (3.6)	0 (0)	
Education, n (%)			0.010^b^
Illiterate	6 (10.9)	20 (36.4)	
Under SSC	25 (45.5)	22 (40.0)	
SSC	16 (29.1)	10 (18.2)	
HSC	8 (14.5)	3 (5.5)	

Current smoking was the most frequent smoking category (30 (54.5%) in the povidone-iodine arm vs. 28 (50.9%) in the salt/soda arm); more than half of the patients had no recorded comorbidity (30 (54.5%) and 33 (60.0%)), and ECOG performance status 0 predominated (37 (67.3%) in the povidone-iodine arm and 36 (65.5%) in the salt/soda arm). Squamous cell carcinoma was the principal histology in both arms (47 (85.5%) and 51 (92.7%)). No significant between-arm differences were found in smoking status (p=0.242), comorbidity (p=0.071), ECOG performance status (p=0.700), primary tumour site (p=0.085), stage (p=0.180), or histology (p=0.221) (Table 2).

**Table 2:** Baseline clinical and tumour characteristics of the study participants by treatment arm (n=110) Data are presented as n (%). A two-sided p<0.05 was considered statistically significant. ^a^Pearson chi-square test ECOG, Eastern Cooperative Oncology Group

Variable	Povidone-iodine (n=55)	Salt/soda (n=55)	p-value
Smoking status, n (%)			0.242^a^
Never	12 (21.8)	19 (34.5)	
Current	30 (54.5)	28 (50.9)	
Former	13 (23.6)	8 (14.5)	
Comorbidity, n (%)			0.071^a^
None	30 (54.5)	33 (60.0)	
Hypertension	25 (45.5)	18 (32.7)	
Cardiovascular disease	0 (0)	4 (7.3)	
ECOG performance status, n (%)			0.700^a^
0	37 (67.3)	36 (65.5)	
1	16 (29.1)	15 (27.3)	
2	2 (3.6)	4 (7.3)	
Primary tumour site, n (%)			0.085^a^
Oral cavity	10 (18.2)	20 (36.4)	
Oropharynx	16 (29.1)	10 (18.2)	
Pyriform fossa	6 (10.9)	11 (20.0)	
Nasopharynx	7 (12.7)	2 (3.6)	
Hypopharynx	2 (3.6)	1 (1.8)	
Larynx (supraglottic/glottic)	14 (25.5)	11 (20.0)	
Stage, n (%)			0.180^a^
II	18 (32.7)	19 (34.5)	
III	33 (60.0)	26 (47.3)	
IV	4 (7.3)	10 (18.2)	
Histology, n (%)			0.221^a^
Squamous cell carcinoma	47 (85.5)	51 (92.7)	
Others	8 (14.5)	4 (7.3)	

Definitive radiotherapy was administered more frequently in the povidone-iodine arm than in the salt/soda arm (47 (85.5%) vs. 37 (67.3%)), whereas postoperative radiotherapy was more frequent in the salt/soda arm (18 (32.7%) vs. 8 (14.5%)); this difference was statistically significant (p=0.025). Radiotherapy-dose distributions were identical (p=1.000). Parallel-opposed fields were used in 51 (92.7%) and 43 (78.2%) of patients in the povidone-iodine and salt/soda arms, respectively, but the difference in field arrangement did not reach statistical significance (p=0.056) (Table 3).

**Table 3:** Radiotherapy characteristics of the study participants by treatment arm (n=110) Data are presented as n (%). A two-sided p<0.05 was considered statistically significant. ^a^Pearson chi-square test ^b^Fisher’s exact test

Variable	Povidone-iodine (n=55)	Salt/soda (n=55)	p-value
Radiotherapy intent, n (%)			0.025^a^
Definitive	47 (85.5)	37 (67.3)	
Postoperative	8 (14.5)	18 (32.7)	
Radiotherapy dose, n (%)			1.000^a^
60 Gy	12 (21.8)	12 (21.8)	
66 Gy	43 (78.2)	43 (78.2)	
Field arrangement, n (%)			0.056^b^
Unilateral single field	4 (7.3)	12 (21.8)	
Parallel-opposed fields	51 (92.7)	43 (78.2)	

WHO grade ≥3 oral mucositis first appeared during week 3. It was significantly more frequent in the povidone-iodine arm than in the salt/soda arm at week 3 (10 (18.2%) vs. 0 (0%); p=0.001), week 4 (14 (25.5%) vs. 4 (7.3%); p=0.010), and week 5 (20 (36.4%) vs. 10 (18.2%); p=0.032). The between-arm differences were not significant at week 6/completion (27 (50.9%) vs. 24 (43.6%); p=0.447) or day 14 after radiotherapy (3 (5.7%) vs. 1 (1.8%); p=0.359). Over the whole period, grade ≥3 mucositis occurred in 33 (60.0%) povidone-iodine patients and 24 (43.6%) salt/soda patients (p=0.086) (Table 4). The corresponding absolute risk differences (salt/soda minus povidone-iodine) were −18.2% (95% CI −28.4 to −8.0) at week 3, −18.2% (95% CI −31.6 to −4.8) at week 4, and −18.2% (95% CI −34.5 to −1.9) at week 5, corresponding to approximately six patients treated for one additional patient free of severe mucositis at each of these weeks. The corresponding risk ratios (salt/soda relative to povidone-iodine) were 0.29 (95% CI 0.10 to 0.81) at week 4 and 0.50 (95% CI 0.26 to 0.97) at week 5; a risk ratio could not be estimated at week 3 because no event occurred in the salt/soda arm. After Bonferroni correction for the five week-specific comparisons (adjusted threshold p<0.010), the week 3 difference remained significant, and the week 4 difference lay at the adjusted threshold, whereas the week 5 difference did not. At week 6/completion, the absolute risk difference was −7.3% (95% CI −26.1 to 11.5) and the risk ratio was 0.86 (95% CI 0.57 to 1.28). Over the whole period, the absolute risk difference was −16.4% (95% CI −34.8 to 2.1) and the risk ratio was 0.73 (95% CI 0.50 to 1.05).

**Table 4:** Incidence of WHO grade ≥3 oral mucositis during radiotherapy and follow-up by treatment arm Data are presented as n (%). A two-sided p<0.05 was considered statistically significant. ^a^Fisher’s exact test. ^b^Pearson chi-square test. ^†^The denominator in the povidone-iodine arm is 53 at these time points because two patients discontinued radiotherapy. At all other time points and for the whole period, the denominator is 55 in both arms. RT, radiotherapy; WHO, World Health Organization

Time point	Povidone-iodine, n (%)	Salt/soda, n (%)	p-value
Week 3	10 (18.2)	0 (0)	0.001^a^
Week 4	14 (25.5)	4 (7.3)	0.010^b^
Week 5	20 (36.4)	10 (18.2)	0.032^b^
Week 6/completion^†^	27 (50.9)	24 (43.6)	0.447^b^
Day 14 after RT^†^	3 (5.7)	1 (1.8)	0.359^a^
Overall (whole period)	33 (60.0)	24 (43.6)	0.086^b^

The incidence of WHO grade ≥3 oral mucositis increased progressively from week 3 to treatment completion in both arms and was consistently lower in the salt/soda arm during this period. The between-arm differences were significant at weeks 3-5, but the proportions converged at completion and declined markedly by day 14 after radiotherapy (Figure 1).

**Figure 1:**
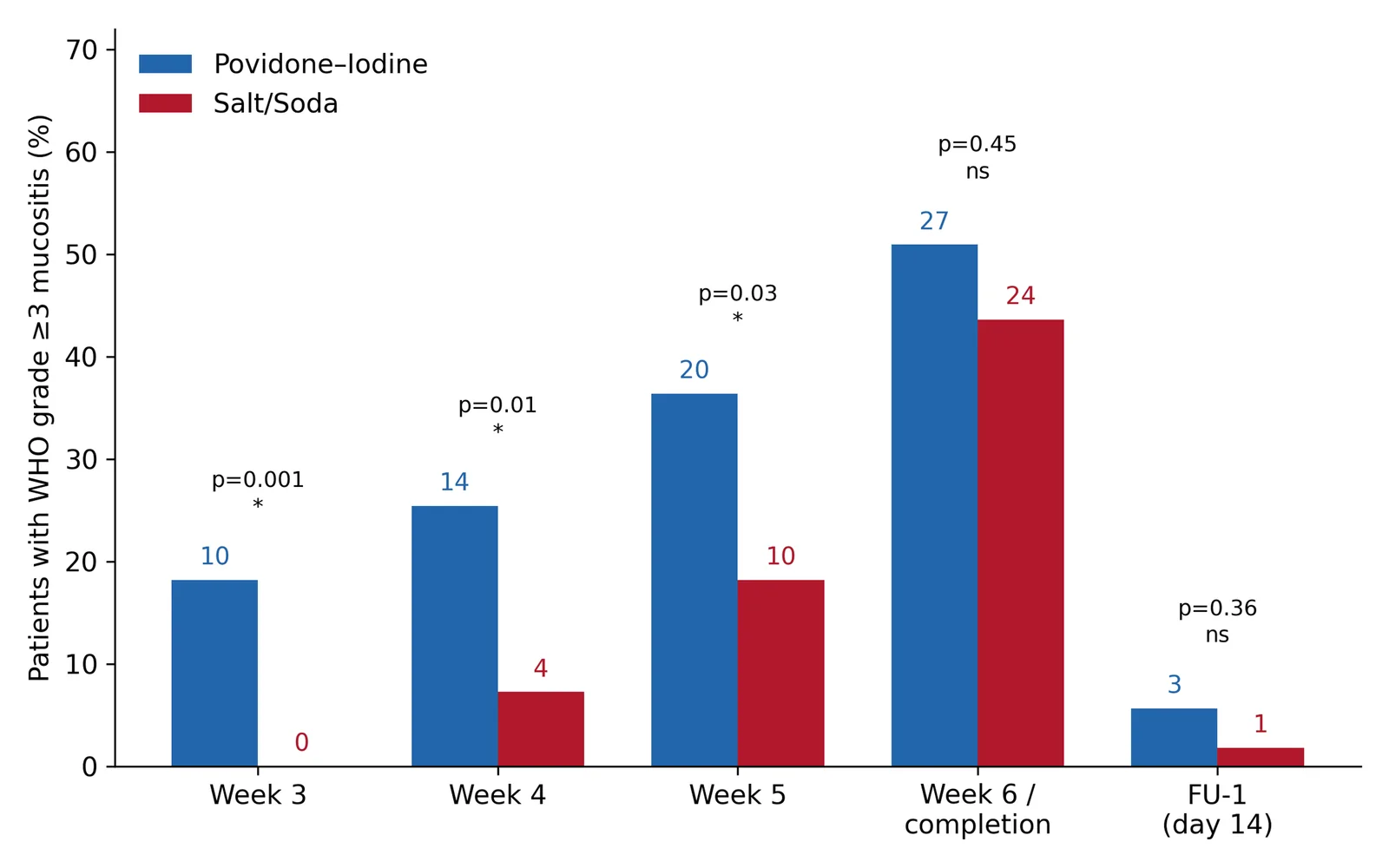
Incidence of WHO grade ≥3 oral mucositis by treatment week in the two arms Bar height shows the percentage of patients with WHO grade ≥3 oral mucositis, and the value above each bar shows the number of patients. The denominator is 55 per arm, except 53 in the povidone-iodine arm at week 6/completion and FU-1 because two patients discontinued radiotherapy. p-values are from the Pearson chi-square or Fisher's exact test. *p<0.05 ns, not significant; FU-1, first follow-up (day 14 after radiotherapy)

The distribution of WHO oral mucositis grades shifted from grades 0-1 toward grades 2-4 as radiotherapy progressed, followed by recovery during follow-up. Grade 1 mucositis and grade ≥3 mucositis each appeared one week later in the salt/soda arm than in the povidone-iodine arm. Grade 4 mucositis first appeared at week 4 in the povidone-iodine arm but only at treatment completion in the salt/soda arm; by day 28 after radiotherapy, no patient in either arm had grade ≥3 mucositis (Figure 2).

**Figure 2:**
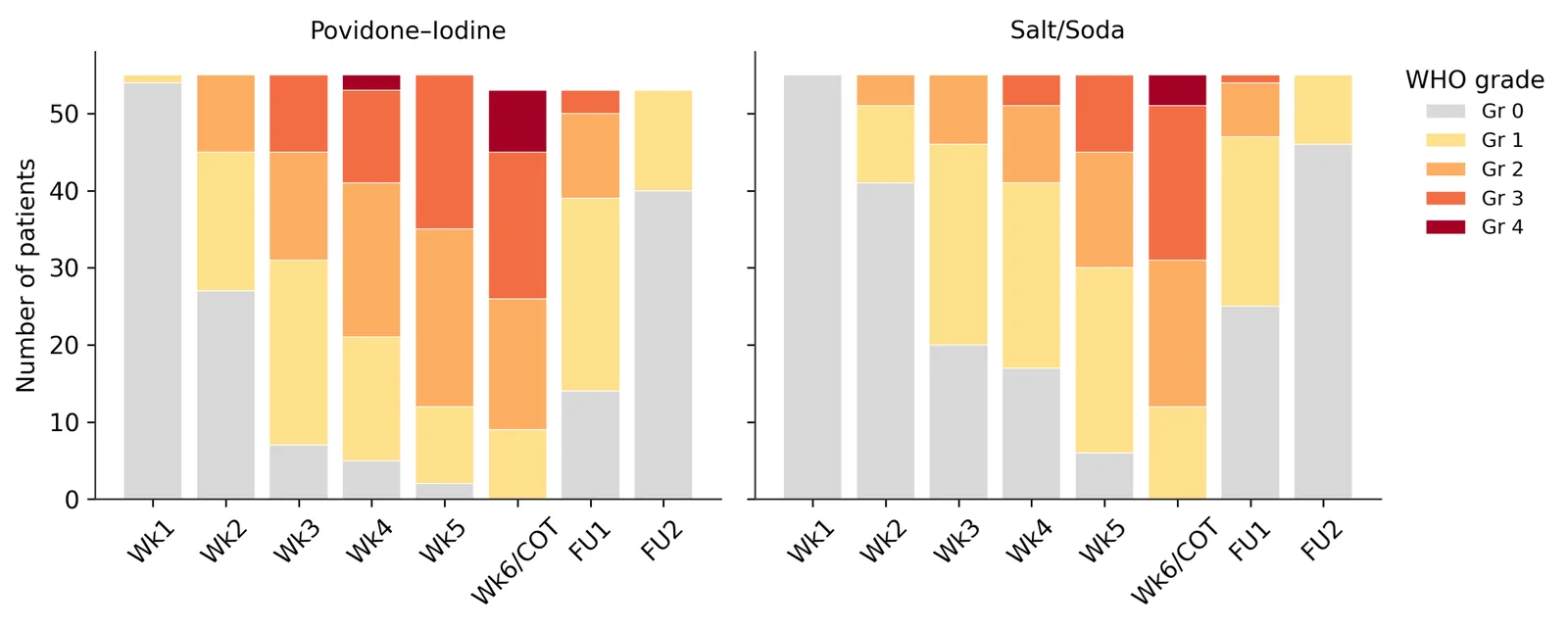
Distribution of all WHO oral mucositis grades (0-4) at each visit for the povidone-iodine (left) and salt/soda (right) arms Data are the number of patients, n (left axis), and the corresponding percentage within each arm (right axis); n=55 per arm, except 53 in the povidone-iodine arm at week 6/COT, FU1, and FU2. Between-arm comparisons are given in Table 4 (two-sided p<0.05 considered significant). COT, completion of treatment; FU, follow-up; Wk, week

Among patients who developed WHO grade ≥3 oral mucositis, the mean duration was 20.71 ± 10.18 days in the povidone-iodine arm (n=31) and 17.21 ± 5.55 days in the salt/soda arm (n=24). The corresponding median durations were 20.0 days (IQR 13.0-24.0) and 16.5 days (IQR 13.0-21.0), respectively, with no statistically significant between-arm difference (p=0.21) (Table 5). The duration of severe mucositis could be determined for 31 of the 33 affected patients in the povidone-iodine arm, because the two patients who discontinued radiotherapy were not followed up to resolution of mucositis; all 24 affected patients in the salt/soda arm were followed up to resolution.

**Table 5:** Duration of WHO grade ≥3 oral mucositis among affected patients by treatment arm Data are presented as means ± standard deviations (SDs) and as median with interquartile range (IQR), in days, among patients who developed WHO grade ≥3 oral mucositis. The denominator in the povidone-iodine arm is 31 rather than 33 because the two patients who discontinued radiotherapy were not followed up to mucositis resolution. p-value is from the independent-samples median test; a two-sided p<0.05 was considered statistically significant. SD, standard deviation; IQR, interquartile range; Q1, first quartile; Q3, third quartile; WHO, World Health Organization

Statistic	Povidone-iodine (n=31)	Salt/soda (n=24)	p-value
Mean ± SD (days)	20.71 ± 10.18	17.21 ± 5.55	0.21
Median (days)	20.00	16.50	
IQR (Q1-Q3)	13.00-24.00	13.00-21.00	

Median patient-reported VAS pain remained 0 through week 2 in both arms and increased one week earlier in the povidone-iodine arm. It reached 2 at week 3 and remained at that level through treatment completion in the povidone-iodine arm; in the salt/soda arm, the median was 0 at week 3, 1 at weeks 4 and 5, and 2 at completion. Median pain returned to 0 in both arms at the day 14 and day 28 follow-up visits (Figure 3). The between-arm differences at week 3 (p=0.001) and week 4 (p=0.006) both remained below the Bonferroni-corrected threshold of p<0.010, although the absolute difference in the median score was one to two points on the 10-cm scale.

**Figure 3:**
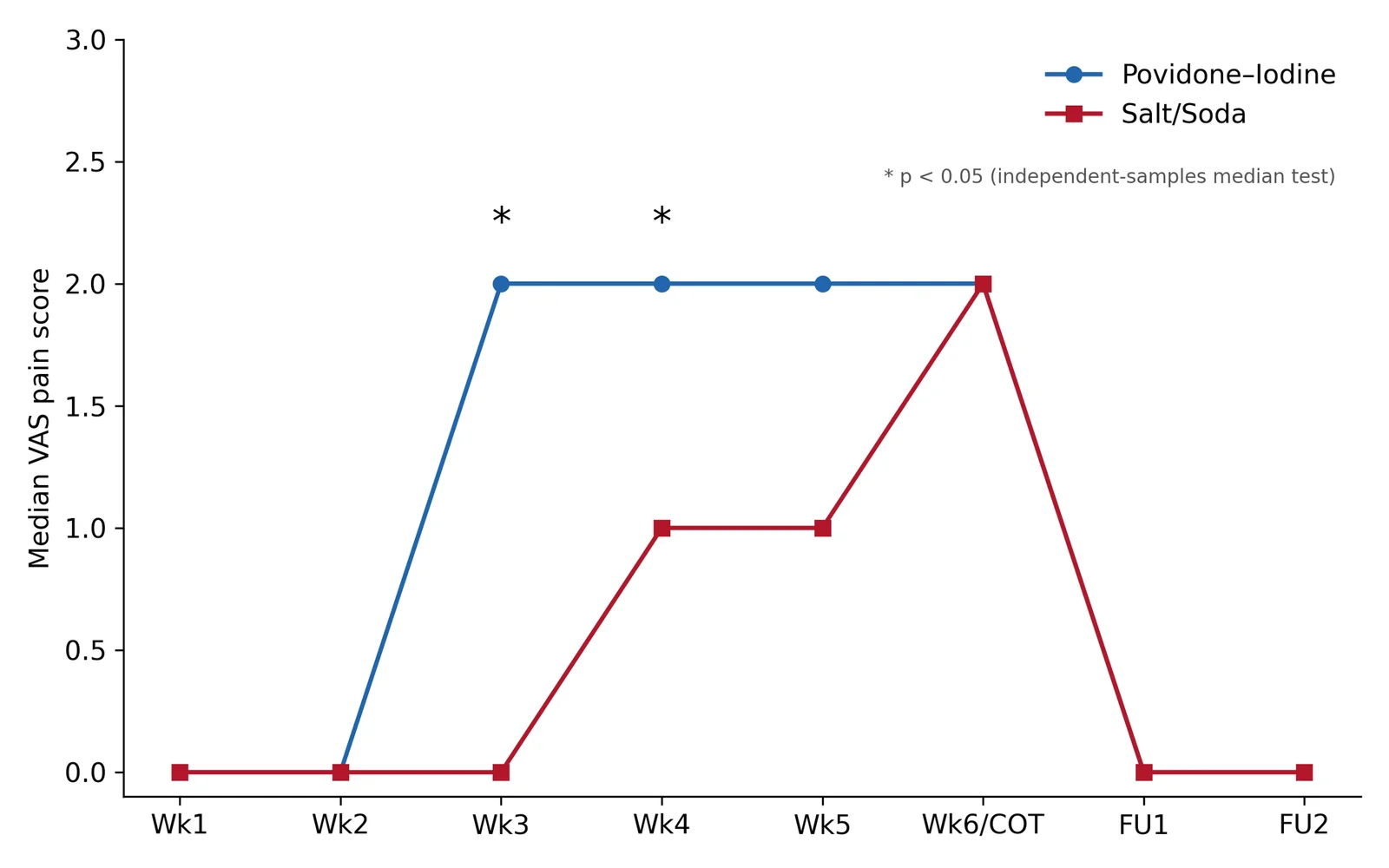
Median VAS pain score over the treatment and follow-up period Data are the median visual analogue scale (VAS) scores (0-10 cm) per arm at each visit (n=55 per arm; n=53 in the povidone-iodine arm at Wk6/COT, FU1, and FU2). Asterisks denote a statistically significant between-arm difference on the independent-samples median test (week 3, p=0.001; week 4, p=0.006), with a two-sided p<0.05 considered significant. COT, completion of treatment; FU, follow-up; Wk, week

Analgesic use did not differ significantly between the povidone-iodine and salt/soda arms (29 (52.7%) vs. 33 (60.0%); p=0.442). Antifungal use was significantly higher in the salt/soda arm than in the povidone-iodine arm (46 (83.6%) vs. 35 (63.6%); p=0.017). Treatment interruption (4 (7.3%) vs. 0 (0%)) and discontinuation (2 (3.6%) vs. 0 (0%)) occurred only in the povidone-iodine arm, although these differences were not statistically significant (p=0.118 and p=0.495, respectively) (Table 6). Expressed as effect sizes (salt/soda relative to povidone-iodine), the absolute risk difference for antifungal use was +20.0% (95% CI 4.0 to 36.0) with a risk ratio of 1.31 (95% CI 1.04 to 1.66), and the absolute risk difference for analgesic use was +7.3% (95% CI −11.2 to 25.8) with a risk ratio of 1.14 (95% CI 0.82 to 1.58). Because treatment interruption and discontinuation occurred only in the povidone-iodine arm, no risk ratio could be estimated. The absolute risk differences (salt/soda minus povidone-iodine) were −7.3% for treatment interruption and −3.6% for treatment discontinuation.

**Table 6:** Analgesic and antifungal use and radiotherapy interruption or discontinuation by treatment arm Data are presented as n (%), with denominators of 55 patients per arm. A two-sided p<0.05 was considered statistically significant. ^a^z-test for proportions. ^b^Fisher's exact test.

Outcome	Povidone-iodine, n (%)	Salt/soda, n (%)	p-value
Analgesic use	29 (52.7)	33 (60.0)	0.442^a^
Antifungal use	35 (63.6)	46 (83.6)	0.017^a^
Treatment interruption	4 (7.3)	0 (0)	0.118^b^
Treatment discontinuation	2 (3.6)	0 (0)	0.495​​​​​​​^b^

## Discussion

The present study indicated a positive trend favouring homemade salt/soda mouthwash during the early weeks of radiotherapy. WHO grade ≥3 oral mucositis was less frequent in the salt/soda arm at weeks 3, 4, and 5; its onset was delayed by about one week, and patient-reported pain was lower at weeks 3 and 4. Over the whole treatment period, however, the incidence and duration of severe mucositis did not differ significantly, and antifungal prescription was more frequent with salt/soda.

The early advantage is consistent with reports on bland bicarbonate-containing rinses, which have been described as delaying the onset of mucositis and reducing the proportion of patients reaching grade 3 or more, and with the earlier randomised comparison of three mouthwashes that informed our sample size, which did not find povidone-iodine superior to a salt/soda rinse [29]. The magnitude of our difference is of a similar order: the largest absolute risk difference was 18.2 percentage points during weeks 3 to 5 (95% CI −28.4 to −8.0 at week 3), with approximately six patients treated for one additional patient free of severe mucositis.

In contrast, a systematic review of sodium bicarbonate-containing rinses found heterogeneous results and did not establish superiority over other agents [22]. Photobiomodulation and benzydamine have stronger contemporary support in selected radiotherapy settings [15,20,21]. The pooled photobiomodulation estimates derive from randomised allocation and are less vulnerable to confounding than ours, whereas the most recent benzydamine study was single-armed and reported compliance rather than an effect size [20,21]. Differences in population characteristics, radiotherapy technique, assessment scales, and background oral care explain much of this heterogeneity. Our whole-period difference of 16.4 percentage points had a confidence interval of −34.8 to 2.1, which is compatible with a benefit, with no difference, and a small disadvantage.

A definitive biological explanation cannot be derived from this study. Sodium bicarbonate can buffer oral acidity and, together with saline, may assist gentle cleansing and the removal of thick secretions, although the clinical efficacy of these mechanisms remains uncertain [22]. Povidone-iodine has broad antimicrobial activity [24], but radiation-induced mucositis is driven by overlapping epithelial, inflammatory, and microbial processes rather than by a single infectious pathway [5,6]. Because salivary pH, microbial composition, and local inflammatory markers were not measured, any mechanistic claim would be speculative, and tolerability, adherence, and baseline imbalance remain equally plausible.

Patient-reported pain paralleled the mucositis pattern, with higher median scores in the povidone-iodine arm at weeks 3 and 4, which is coherent because ulcerative mucositis is a major source of oral pain [11,12]. As pain is subjective and the study was unblinded, it was analysed as a secondary patient-reported outcome, and is best read as a supportive trend. The higher use of nystatin in the salt/soda arm (83.6% vs. 63.6%; absolute risk difference 20.0 percentage points, 95% CI 4.0 to 36.0) requires separate consideration. Candidiasis was not confirmed microbiologically, so antifungal prescription was a management outcome rather than evidence that salt/soda increased fungal infection, and the antimicrobial activity of povidone-iodine may have influenced prescribing [24,27]. Radiotherapy interruption (7.3% vs. 0%) and discontinuation (3.6% vs. 0%) occurred only with povidone-iodine, with too few events for a reliable estimate.

Systemic iodine exposure is a further distinction. Intra-oral povidone-iodine can raise serum and urinary iodine, and repeated rinsing may produce ingestion above the recommended intake [25,26]. Thyroid function and iodine status were not measured here, and adverse effects of the rinses themselves were not systematically recorded, so no comparative safety conclusion can be drawn. From a service perspective, the salt/soda ingredients are inexpensive, locally available, and the wash can be prepared at home, which may matter where commercial rinses or photobiomodulation are inaccessible, although affordability does not establish first-line effectiveness.

The present study has several strengths. To the best of our knowledge, it is the first direct head-to-head comparison of povidone-iodine and homemade salt/soda mouthwash for the prophylaxis of radiation-induced oral mucositis in a South Asian setting; it collected data prospectively at seven pre-specified time points, and reports its non-random allocation and unadjusted analysis transparently. However, our study had several limitations. Individual allocation followed a predictable odd/even sequence and recruitment was by convenience sampling, so selection bias cannot be excluded. The arms were imbalanced in socio-economic status, education, and radiotherapy intent, and with 110 patients and 57 events, the data could not support stable adjustment for indication-dependent confounding or trend modelling. Every comparison is therefore unadjusted. The week-specific tests were not planned as a family, and after Bonferroni correction, only the week 3 and week 4 differences remained at or below the adjusted threshold; hence, these multiple unadjusted comparisons and the baseline imbalances may account for part of the early difference. The radiotherapy modality was uniform, but two-dimensional treatment irradiates a wide mucosal volume to a high dose and yields no mucosal dose-volume histogram, so the findings should not be extrapolated to IMRT or VMAT practice. Blinding was not feasible, mucositis was graded by a single unblinded investigator without duplicate assessment, adherence was not monitored, and background oral care was neither standardised nor recorded. Patients on concurrent chemoradiation were excluded, and the study was not powered for the secondary outcomes. The findings should therefore be regarded as a hypothesis-generating trend rather than a causal or comparative-effectiveness claim.

## Conclusions

In this study, a positive trend favouring salt/soda mouthwash was observed during early radiotherapy, with the incidence of severe RIOM and patient-reported oral pain levels lower than those observed with povidone-iodine, whereas the overall incidence and duration of severe mucositis did not differ significantly between the two rinses. Because individual allocation was predictable, the arms were imbalanced at baseline, all comparisons were unadjusted, and the week-specific tests were multiple, these findings describe an exploratory association and not a demonstrated comparative effect. Because it is inexpensive, can be prepared at home, and does not entail iodine exposure, salt/soda mouthwash may nevertheless be a practical prophylactic option in resource-limited settings. Adequately powered randomized multicentre trials, with concealed allocation and blinded outcome assessment, including patients receiving concurrent chemoradiation and patients treated with modern conformal techniques, are needed before definitive comparative-effectiveness or first-line recommendations can be made.
